# Supplementary material for: Impact of ABC Transporters in Osteosarcoma and Ewing’s Sarcoma: Which Are Involved in Chemoresistance and Which Are Not?
Source: Cells. 2021 Sep 17;10(9):2461. doi: 10.3390/cells10092461 (PMC8467338; doi:10.3390/cells10092461)
Supplement: Supplementary file 1 [file cells-10-02461-s001.zip › cells-1300589-supplementary.pdf]

Supplementary Table S1. Summary of role(s) of the ABC transporters discussed in this review for osteosarcoma and/or Ewing's sarcoma. Data concerning germline single nucleotide polymorphisms were not considered because they are summarized in Table 1.

ABC transporter	*Role(s) in OS and/or ES*	*Reference*
ABCA1	Downregulation associated with:<br>- doxorubicin resistance of OS cells<br>- decreased recognition by Vγ9Vδ2 T-lymphocytes<br>- drug resistance of CSCs	 [42]<br>[42]<br>[84; 88]
ABCA2	- Overexpression in OS CSCs that are more resistant to chemotherapeutic drugs	[81; 84]
ABCA3	- Presence in OS CSCs that are more resistant to chemotherapeutic drugs	[40]
ABCA5	- Presence in OS CSCs that are more resistant to chemotherapeutic drugs<br><br>- Involvement in the pro-metastatic phenotype of OS CSCs<br><br>- Higher expression in lung metastases compared to primary OS<br><br>- Differential expression associated with stemness properties of OS cells	[41]<br><br>[41]<br><br>[41]<br><br>[83]
ABCA6	- Increased expression associated with favourable prognosis in ES	[56]
ABCA7	- Increased expression associated with favourable prognosis in ES	[56]
ABCA8	- Differential expression associated with	[83]

	stemness properties of OS cells	
ABCA9	- Involved in drug resistance of OS CSCs	[88]

ABC transporter	*Role(s) in OS and/or ES*	*Reference*
ABCB1	Increased expression associated with:<br>- doxorubicin resistance in OS<br>- unresponsiveness to other drugs used in first-line or rescue OS treatments<br>- worse clinical outcome<br><br>- Overexpression in OS CSCs that are more resistant to chemotherapeutic drugs<br><br>- Differential expression associated with stemness properties of OS cells<br><br>- Discrepant results concerning the impact on clinical outcome in ES<br><br>- Increase of ES drug resistance through an efflux activity induced by GLI1<br><br>- Overexpression associated with enhanced clonogenicity, invasiveness and resistance against cisplatin and doxorubicin in ES side population cells	 [10; 12; 42]<br>[11; 12]<br><br>[13; 33]<br><br>[81; 84; 85; 87; 88]<br><br><br>[83]<br><br><br>[48-54]<br><br><br>[66]<br><br><br>[94]
ABCB2	- Increased ES drug resistance through an efflux activity induced by GLI1	[66]
ABCB5	- Overexpression in OS CSCs that are more resistant to chemotherapeutic drugs	[87]
ABCB10	- Differential expression associated with stemness properties of OS cells	[83]
ABCC1	- Increased expression associated with drug resistance in OS<br><br>- Increased expression associated with stemness properties of OS cells	[12]<br><br><br>[83]

	- High levels on mitochondria in drug-resistant ES cells	[60, 61]
ABCC4	- Involvement in doxorubicin resistance of OS cells<br><br>- Differential expression associated with stemness properties of OS cells	[18]<br><br>[83]

ABC transporter	*Role(s) in OS and/or ES*	*Reference*
ABCC5	- Increased expression associated with worse EFS in OS<br><br>- Differential expression associated with stemness properties of OS cells	[19]<br><br>[83]
ABCC6	- Differential expression associated with stemness properties of OS cells	[83]
ABCD3	- Differential expression associated with stemness properties of OS cells	[83]
ABCF1	Low expression of ABCF1, coupled with IGF2BP3 high levels, associated with worse prognosis	[57]
ABCG2	- Involvement in OS drug resistance<br><br>- Differential expression associated with stemness properties of OS cells<br><br>- Overexpression in OS CSCs that are more resistant to chemotherapeutic drugs<br><br>- Increased expression associated with worse survival in ES<br><br>- Increase of ES drug resistance through an efflux activity induced by GLI1<br><br>- Overexpression associated with enhanced clonogenicity, invasiveness and resistance against cisplatin and doxorubicin in ES side population cells	[20]<br><br>[82, 83]<br><br>[81; 84; 85; 87]<br><br>[55]<br><br>[66]<br><br>[94]

Legend: CSCs, cancer stem cells; EFS, event-free survival; ES, Ewing's sarcoma; GLI1, glioma-associated oncogene homolog 1; IGF2BP3, insulin like growth factor 2 mRNA binding protein 3; OS, osteosarcoma.
